# Knee-Deep in Bias: When the Low-Probability Diagnosis of Double-Expressor Lymphoma Wins

**DOI:** 10.7759/cureus.103817

**Published:** 2026-02-18

**Authors:** Devin C Weber, James M Day

**Affiliations:** 1 Flight Surgery, US Navy, Yigo, USA; 2 Internal Medicine, Naval Medical Center Portsmouth, Portsmouth, USA; 3 Internal Medicine, Naval Hospital Guam, Agana, USA

**Keywords:** austere environment, dlbcl, double expressor lymphoma, gout, knee pain, military medicine, pola-r-chp, resource disparities within cnmi

## Abstract

In resource-constrained, geographically isolated regions like the Commonwealth of the Northern Mariana Islands (CNMI), diagnostic delays can have profound consequences, particularly for aggressive lymphomas. This case report details the journey of a 32-year-old female originally from Tinian, whose initial presentation of knee pain was attributed to gout, masking an underlying double expressor subtype of diffuse large B-cell lymphoma. The limitations in CNMI, including the absence of MRI, prolonged referral pathways, a lack of specialists, and logistical hurdles in obtaining specialized molecular diagnostics, significantly delayed accurate diagnosis. Over months, her condition progressed to include lytic bone lesions, soft tissue masses, and ultimately, widespread systemic involvement requiring definitive management at a higher-level facility. The case underscores how resource disparities in remote settings can obscure rare diagnoses, emphasizing the need for heightened clinical suspicion, innovative diagnostic strategies, streamlined referral processes, and/or expansion of virtual healthcare to improve outcomes in such vulnerable populations.

## Introduction

Double expressor lymphoma (DEL) represents an aggressive subset of diffuse large B-cell lymphoma (DLBCL), characterized by the immunohistochemical overexpression of MYC and BCL2 proteins without corresponding gene rearrangements [1-2]. DEL accounts for approximately 20%-30% of DLBCL cases and generally carries a poorer prognosis compared to non-DEL DLBCL, although its outlook is less dismal than that of double-hit lymphomas involving genetic rearrangements [3]. The germinal center B-cell (GCB) phenotype is confirmed through immunophenotypic markers and molecular profiling, with these lymphomas frequently presenting in advanced stages and involving extranodal sites such as bone and synovium [4].

Diagnosing DEL can be particularly challenging in remote and resource-limited settings, such as the Commonwealth of the Northern Mariana Islands (CNMI), where access to advanced diagnostic tools, including molecular testing and specialized pathology, is often limited. Patients in such regions often experience delays stemming from geographic isolation, restricted access to subspecialty care, and logistical hurdles related to specimen transport and referral processes. These barriers can result in patients presenting with more advanced disease and facing poorer treatment outcomes [1,3].

In cases of monoarticular joint pain such as knee discomfort, initial assessments usually prioritize common etiologies-including gout, osteoarthritis, bursitis, tendinopathies, and septic arthritis-due to their higher pretest probabilities [5]. Gout, for instance, is highly prevalent among Pacific Islander populations and often occupies a prominent differential diagnosis in cases of acute monoarthritis [6]. Diagnostic workup in these scenarios typically begins with clinical evaluation and basic laboratory tests, with advanced imaging and tissue biopsy reserved for cases that do not respond to initial management or remain unexplained. The probability of diagnosing lymphoma is initially low; thus, it is generally considered after more common conditions have been excluded, and standard treatments have failed [5,7].

Accurate diagnosis of DEL demands immunophenotypic and molecular assessment, as recommended by the National Comprehensive Cancer Network [8]. Delays in diagnosis are often intensified by the need for specialized testing, emphasizing the importance of maintaining a broad differential and conducting comprehensive evaluations in patients with persistent, unexplained musculoskeletal symptoms. This case underscores the challenges faced in resource-constrained environments and highlights the critical role of interdisciplinary collaboration in diagnosing rare yet aggressive malignancies like double expressor subtype DLBCLs [3].

## Case presentation

We present an unfortunate case of a 32-year-old female military dependent with a past medical history significant for alcohol dependence who ultimately was diagnosed with stage IV DLBCL. This patient’s story began six months before diagnosis with the onset of left knee pain, recognized as an acute gout flare by the Emergency Department in Tinian. Physical exam revealed generalized swelling about the knee, palpable heat, and tenderness to palpation of the medial and lateral joint lines. Initial lab workup revealed a mildly elevated uric acid level of 6.1 mg/dL (2.4-6.1 mg/dL) and C-reactive protein (CRP) of 5.03 mg/dL (0-0.3 mg/dL). Knee radiographs were unremarkable. Initial management included naproxen and colchicine. A follow-up primary care visit included additional serum tests that resulted in negative antinuclear antibodies (ANAs) and rheumatoid factor. The diagnosis of acute gout was supported, albeit without offering joint aspiration at this time, and allopurinol was added to her medication list. Pain temporarily improved with these medications; however, it ultimately returned two months before the neoplastic diagnosis. The patient traveled to Saipan to be near her husband and established care with a new primary care physician (PCP), who advised twice-daily naproxen and joint aspiration, which she deferred. After one month of twice-daily naproxen, she returned with worsening pain. Physical examination revealed tenderness to palpation, warmth, swelling, and an approximately 4-cm lesion of the proximal medial tibia. This lesion was thought to represent possible tophi. At that time, she was started on a prednisone taper and continued on naproxen, and repeat radiographs were obtained. These radiographs demonstrated a permeative sclerotic process of the proximal tibia. This was a significant turning point in the diagnostic workup as other pathologies were considered. One week later, she returned for ultrasound-guided knee joint aspiration, but the procedure was unsuccessful. The performing physician ordered an outpatient CT scan of the knee for further evaluation, as an MRI was not available on Saipan.

**Figure 1:**
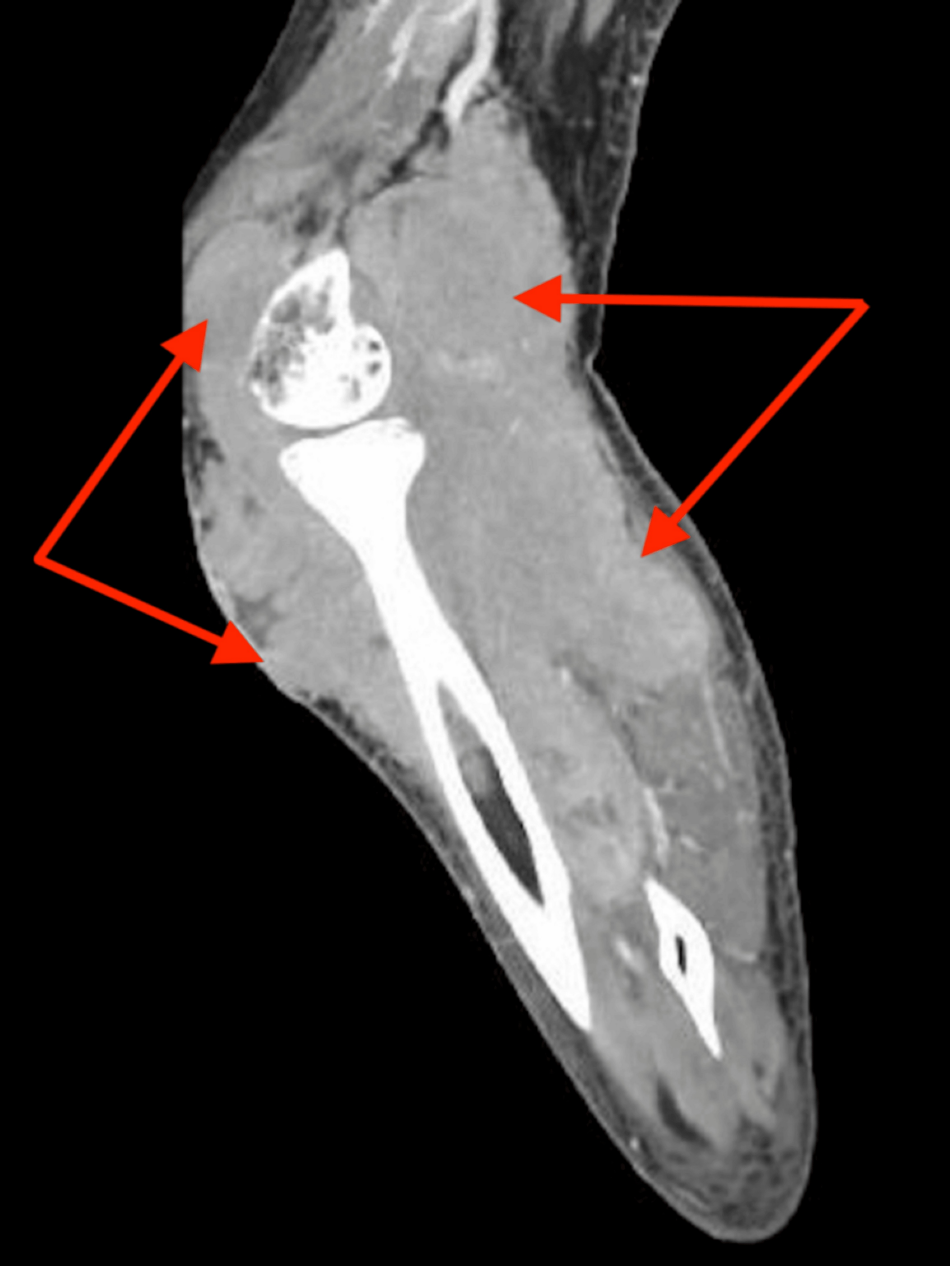
Large mass within the region of the gastrocnemius measuring 9 × 8 cm, and a large heterogeneous mass-like area involving the left pretibial musculature measuring 8 × 4 cm at the greatest dimension (arrows).

**Figure 2:**
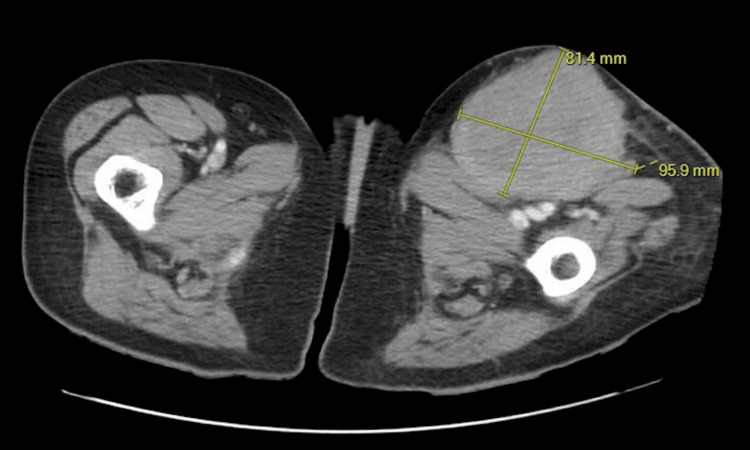
A 9.6 × 8.1 cm soft-tissue mass in the left superior anterior thigh with enlarged left external iliac lymph nodes.

The patient presented to the Emergency Room (ER) one week after the failed knee aspiration secondary to a newly discovered tender left-sided inguinal mass. A left lower extremity ultrasound revealed large left inguinal and popliteal soft-tissue masses consistent with pathologic lymph nodes. A CT scan obtained in the emergency department, as seen in Figure 1, revealed multiple masses about the left knee and left calf with significant heterogeneity and bony involvement. Figure 2 demonstrates CT imaging of an ipsilateral proximal thigh mass with associated pelvic lymphadenopathy. Radiology was consulted for an expedited CT-guided biopsy of the inguinal mass, and Orthopedics was consulted for surgical evaluation. The inguinal biopsy was performed the following day, followed by a partial excisional biopsy of the knee mass within five days. She was prescribed Percocet for postoperative analgesia.

Given resource limitations within the CNMI, the patient was transported to Guam to complete an MRI of the left knee and to establish command sponsorship within the military health care system. 10 days status post orthopedic surgery, she developed a thrombus in her left tibial veins and was started on Apixaban twice daily. Two weeks later, she had a command sponsorship appointment with her new PCP, at which time immunohistochemistry of the inguinal mass returned with a pathological diagnosis of large B-cell lymphoma showing positive CD20 and Ki-67 elevated at 90%. Her PCP addressed her pain with as-needed Celebrex and extended-release Morphine, but she ended up returning to the ER two weeks later with intractable left leg pain and swelling. Repeat ultrasound redemonstrated a large infiltrating mass completely occluding the left popliteal vein. She had seen her oncologist just one week prior, who recommended off-island movement to Hawaii for treatment, given the unusual presentation of her lymphoma with aggressive features. Given her inadequate pain control on outpatient analgesia in the setting of a new malignancy, she was admitted to the hospital, and expedited cancer staging commenced.

**Figure 3:**
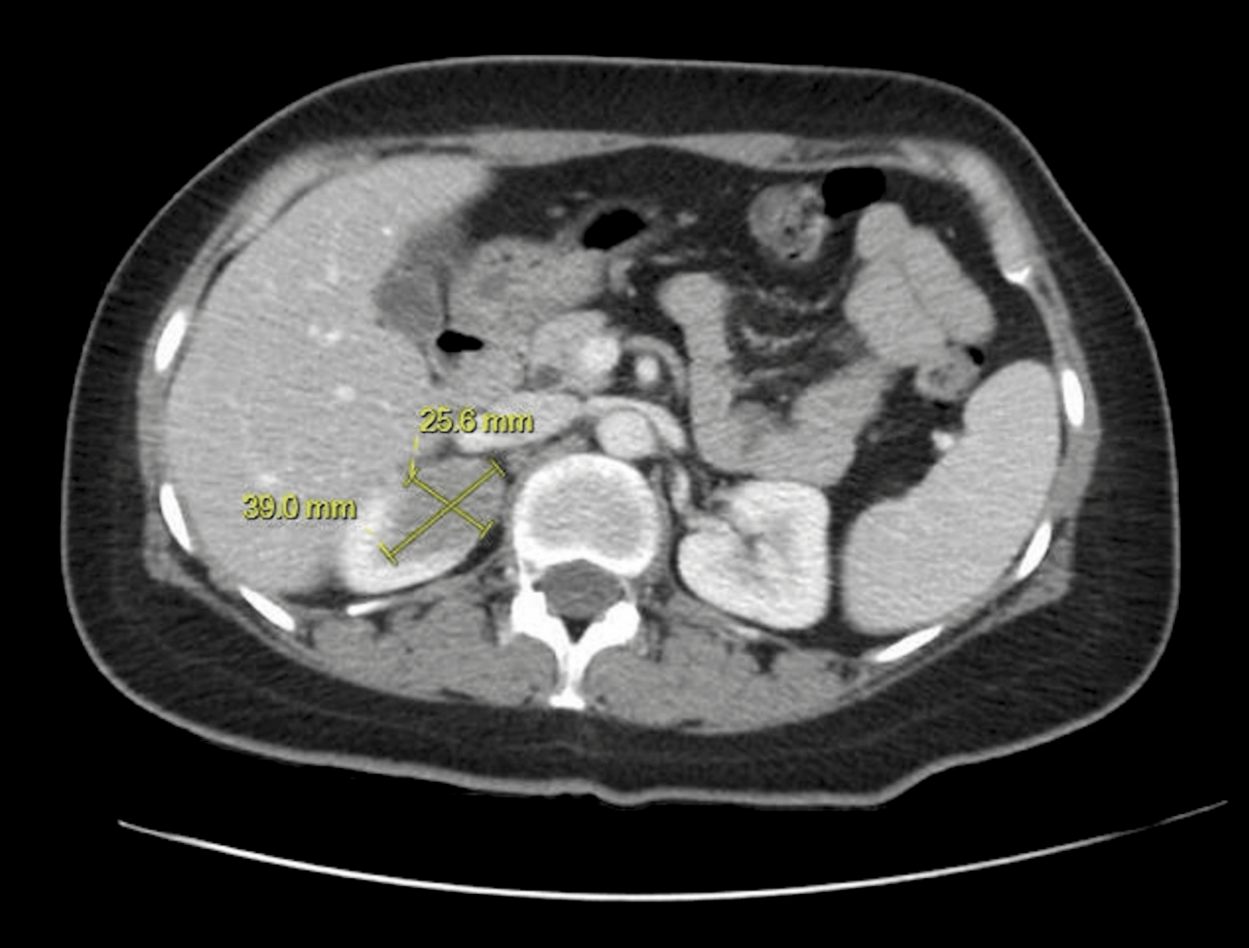
A 3.9 × 2.6 cm soft-tissue mass in the superior pole of the right kidney.

Chest, abdomen, and pelvis CT imaging revealed the following: a soft-tissue mass in the superior pole of the right kidney (Figure 3); enlarged left external iliac lymph nodes; left-sided subcutaneous edema extending from the level of the kidneys to the left thigh, consistent with anasarca; and a hemangioma in the left hepatic lobe. A dedicated MRI of the left knee showed a very large (at least 18.6 cm in the craniocaudal dimension), infiltrative, lobulated soft-tissue mass centered at the knee, involving the subcutaneous fat, muscles, and joint space. An abnormal signal in the tibia and superior fibula was compatible with mass infiltration. After a four-day hospitalization, she was transported to Tripler Army Medical Center (TAMC) in Hawaii for definitive management.

**Figure 4:**
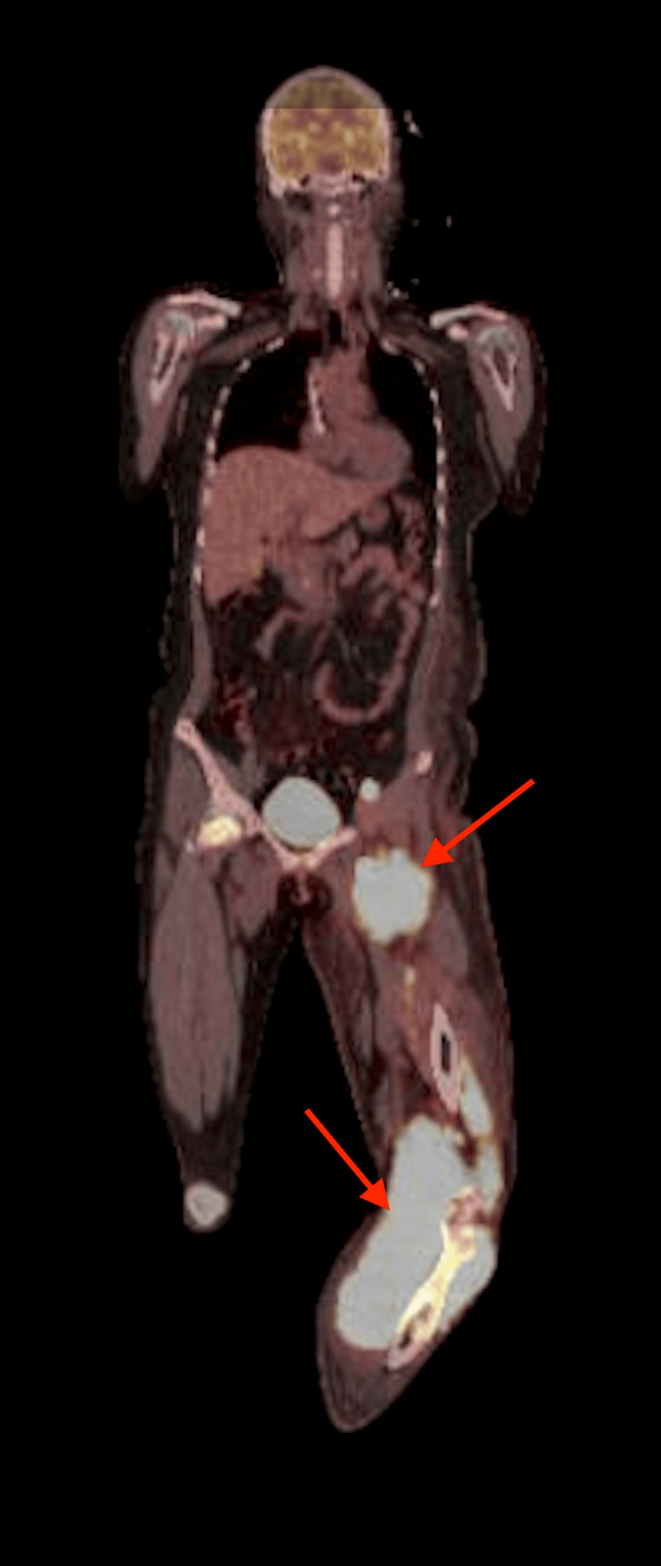
PET/CT showing intense radiotracer uptake within a left anterior thigh soft-tissue density mass measuring 7.5 × 9.4 cm and a left knee multicompartment soft-tissue density mass measuring 13.6 × 10.2 cm (arrows). PET/CT, positron emission tomography/computed tomography

Before systemic chemotherapy was initiated, oncology recommended repeat tibial bone biopsies and a positron emission tomography (PET) scan to ascertain the full extent of the disease. Fluorescence in situ hybridization (FISH) analysis of the bone biopsy confirmed double-expressor germinal center B-cell lymphoma. PET CT scan, completed shortly after inpatient transport, identified multiple areas of radiotracer uptake beyond the initial focus, including the gray-white matter junctions throughout the brain; the region of the splenic vein near the pancreatic tail; the left anterior thigh; the left external iliac chain lymph nodes; the right superior pole renal mass; the right femoral head; and the left mandible. Radiotracer uptake within the inguinal and knee masses is portrayed in Figure 4. Brain uptake upstaged her Central Nervous System International Prognostic Index score to high risk. Lumbar puncture to rule out central nervous system (CNS) involvement was deferred before the initiation of chemotherapy because of ongoing oral anticoagulation and the patient’s clinical status (pain and compromised neurovascular anatomy of the left lower extremity with persistent neuropathy), so as not to delay cycle 1 of polatuzumab vedotin plus rituximab, cyclophosphamide, doxorubicin, and prednisone (Pola-R-CHP). The proposed management plan was six cycles every 21 days. While at TAMC, she had elevated uric acid levels, most likely due to autolysis from high tumor burden, and was managed with IV fluids and Febuxostat. She began prophylaxis with valacyclovir, trimethoprim-sulfamethoxazole (TMP-SMX), a proton pump inhibitor (PPI), and granulocyte colony-stimulating factor (G-CSF) on day 2 of each cycle.

After spending an additional 10 days in the hospital, she was discharged with oncology shifting focus to the outpatient setting. CNS involvement rule-out was executed after completing cycle 1. Magnetic resonance imaging/magnetic resonance angiography (MRI/MRA) of the brain was largely unremarkable. Lumbar puncture was performed with the patient off anticoagulation for a short window without identification of malignant cells. The patient tolerated cycles 2-6 without reports of adverse effects. A repeat PET scan after cycle 4 demonstrated a significant interval reduction in the size and fluorodeoxyglucose (FDG) avidity of the left lower extremity masses and near-complete resolution of hypermetabolic activity within the abdominal and pelvic masses and lymphadenopathy. The end-of-treatment PET scan showed a similarly appearing anterior thigh mass with a Deauville score of 3, and oncology suspected complete remission. 

## Discussion

The diagnosis of DEL DLBCL in this young adult was significantly delayed, largely because lymphoma is rarely considered in the differential diagnosis of knee pain in this context. In the CNMI and similar Pacific Island settings, acute or chronic knee pain is predominantly attributed to gout, osteoarthritis, trauma, or infectious causes-conditions that are far more prevalent given the high rates of gout and metabolic diseases among Pacific Islanders [5-6]. Primary lymphomas involving bone or synovium are exceedingly rare and are typically not suspected until more common etiologies are excluded or initial treatments prove ineffective [9].

The healthcare infrastructure in the CNMI faces significant challenges driven by geographic isolation, limited resources, and underdeveloped medical facilities [10]. Critical diagnostic tools-advanced imaging modalities, specialized pathology, and molecular testing-are often inaccessible locally, hindering accurate classification and risk stratification of aggressive lymphomas like DEL in a timely manner. Many Pacific island jurisdictions lack in-country specialists, requiring off-island referral for confirmatory diagnosis and treatment, which introduces logistical delays and additional costs [11]. Equipment failures and disruptions in supply chains further exacerbate these barriers [12].

In non-military populations, these barriers are even more pronounced. The military healthcare system offers streamlined access to referrals, transportation, and specialist care, contrasting with fragmented civilian health systems that often rely on limited public resources and complex referral pathways. Financial constraints, travel distances, and lack of insurance coverage commonly delay presentation, leading to diagnoses at more advanced disease stages and poorer outcomes [13]. Data from the Pacific Regional Central Cancer Registry indicate that over 70% of adult cancers in US-affiliated Pacific Islands are diagnosed at stage III or later, with five-year survival rates significantly diminished in some regions, ranging between 10% and 20%, highlighting the impact of delayed diagnosis and limited access to care [12]. 

## Conclusions

This case serves as a sobering reminder that in remote or resource-limited environments, a provider's most effective tool is the willingness to pause and re-evaluate. When standard treatments for a common condition like gout fail to provide lasting relief, or symptoms return shortly after initial therapy, we must be disciplined enough to step back and question our initial assumptions. In regions like the CNMI, where geographic isolation and a lack of advanced imaging already create significant hurdles, maintaining a high index of suspicion for rare malignancies is a necessity, not a luxury.

By improving referral pathways and integrating virtual healthcare, we can help bridge the gap between isolated clinics and the life-saving diagnostics available at larger centers. While this patient ultimately reached a higher echelon of care, her journey highlights the narrow margin for error when dealing with aggressive lymphomas. For those practicing in the field, the takeaway is clear: when a patient is not getting better, the most dangerous move is to remain fixed on a diagnosis that no longer fits the clinical picture.
